# Exploring the impact of rehabilitation on post-surgical recovery in elbow fracture patients: a cohort study

**DOI:** 10.1007/s12306-024-00848-8

**Published:** 2024-07-18

**Authors:** D. Donati, S. Aroni, R. Tedeschi, S. Sartini, G. Farì, V. Ricci, F. Vita, L. Tarallo

**Affiliations:** 1 Physical Therapy and Rehabilitation Unit, Policlinico di Modena, Modena, Italy; 2 Clinical and Experimental Medicine PhD Program, University of Modena and Reggio Emilia, Modena, Italy; 3 Studio Aroni, Private Practice, Bologna, Italy; 4 Department of Biomedical and Neuromotor Sciences (DIBINEM), Alma Mater Studiorum University of Bologna, Via Zamboni 33, 40126 Bologna, Italy; 5 Physical Therapy and Rehabilitation Uniti, Istituto Ortopedico Rizzoli, Argenta, Italy; 6 Department of Experimental Medicine (Di.Me.S.), University of Salento, Lecce, Italy; 7 Physical and Rehabilitation Medicine Unit, Luigi Sacco University Hospital, 20121 Milano, Italy; 8 IRCCS Istituto Ortopedico Rizzoli, 1st Orthopaedics and Traumatology Clinic, Bologna, Italy; 9 Department of Orthopedics and Traumatology, Polyclinic of Modena, University of Modena and Reggio Emilia, Modena, Italy

**Keywords:** Elbow fractures, Rehabilitation, Post-surgical recovery, Joint range of motion (ROM), Individualized care

## Abstract

**Introduction:**

Elbow fractures, characterized by their complexity, present significant challenges in post-surgical recovery, with rehabilitation playing a critical role in functional outcomes. This study explores the efficacy of rehabilitative interventions in enhancing joint range of motion (ROM) and reducing complications following surgery for both stable and unstable elbow fractures.

**Methods:**

A cohort of 15 patients, divided based on the stability of their elbow fractures and whether they received post-operative rehabilitation, was analyzed retrospectively. Measurements of ROM—including flexion, extension, pronation, and supination—were taken at three follow-ups: 15-, 30-, and 45-day post surgery. The study assessed the impact of rehabilitation on ROM recovery and the resolution of post-surgical complications.

**Results:**

The findings indicated no statistically significant differences in ROM improvements between patients who underwent rehabilitation and those who did not, across all types of movements measured. However, early rehabilitative care was observed to potentially aid in the mitigation of complications such as joint stiffness, especially in patients with stable fractures.

**Conclusion:**

While rehabilitation did not universally improve ROM recovery in elbow fracture patients, it showed potential in addressing post-operative complications. The study underscores the importance of individualized rehabilitation plans and highlights the need for further research to establish evidence-based guidelines for post-surgical care in elbow fractures.

## Introduction

The approach to post-surgical rehabilitation of traumatic elbow pathologies stands as one of the most intricate and challenging domains within orthopedic and rehabilitative practice. The scientific literature highlights a wide array of treatments and rehabilitative protocols, reflecting a landscape where consensus on optimal methodologies remains elusive [1–3]. The anatomical and functional complexity of the elbow, coupled with the diversity of injuries and individual patient needs, demands an evidence-based, personalized approach, aiming ultimately at the restoration of joint functionality and the reduction of recovery times [4, 5]. Despite technological advancements and a deeper understanding of bone and tissue healing mechanisms, the management of the immediate post-surgical period, particularly regarding immobilization times and the initiation of rehabilitative treatments, remains an area of intense investigation and debate [6–8]. The emerging need is thus twofold: On one hand, there is a necessity to establish clear, evidence-based guidelines for post-surgical elbow rehabilitation protocols; on the other hand, addressing the knowledge gap regarding the specific influence of immobilization times on functional recovery is imperative [9–12]. In response to this need, the present study proposes to investigate, through a longitudinal observational design, the effectiveness of different rehabilitative approaches in the context of the immediate post-surgical period for elbow fractures. Starting from the hypothesis that timely rehabilitative intervention may promote superior functional outcomes, this research aims to provide concrete evidence on the impact of immobilization times and the optimal sequence of rehabilitative interventions [13–15]. Through a detailed analysis of 15 clinical cases, the study focuses on observing functional progress, assessed by joint range of motion (ROM) and the presence of complications, in relation to the promptness and specificity of rehabilitative interventions. Hence, the inquiry not only addresses the need for clarification in the post-surgical rehabilitative landscape of the elbow but also aspires to make a significant contribution to the scientific literature, offering insights for a more informed and patient-oriented clinical practice [9]. The goal is twofold: to define rehabilitative strategies that can accelerate functional recovery and to outline a clinical pathway that minimizes the risk of complications, thereby optimizing patient outcomes [3, 16, 17]. Ultimately, the contribution of this research fits within the broader context of evidence-based medicine, laying the groundwork for future investigations and a more targeted and specific approach in the rehabilitation of traumatic elbow pathologies.

## Material and methods

The study aimed to explore the treatment responses for traumatic pathologies treated surgically through monitoring in the immediate post-operative phase, investigating the influence of immobilization times on rehabilitative treatments. Therefore, this study sought to demonstrate that rehabilitation proposed in the first 6-week post surgery (during the initial rehabilitative phase) leads to better patient recovery in terms of time and functionality. Patients operated on for elbow pathologies were followed using an attached form (the form below), categorized by pathology (stable and unstable fracture) while considering immobilization times. A follow-up was conducted at 15, 30, and 45 days at clinics dedicated to the upper limb. This allowed for the study of recovery times to identify those who managed to restore function through an invitation to spontaneous mobilization or those who began with physiotherapeutic rehabilitation early.

To systematically capture the outcomes of post-surgical treatments for traumatic elbow pathologies, the study employed a structured follow-up form that meticulously recorded each participant’s progression. This comprehensive approach was pivotal in discerning the efficacy of the physical therapy interventions applied and their impact on the rehabilitation process.

### Physical therapy intervention

The initial section of the form meticulously categorizes patients based on whether they received physical therapy (Yes/No). This binary classification allows for a direct comparison of recovery trajectories between those who underwent formal rehabilitation programs and those who did not, providing a clear demarcation for subsequent analysis.

### Recovery of joint range of motion (ROM)

To quantify the functional outcomes of the treatments, the form includes detailed fields for recording the degrees of recovered motion in four critical movements: flexion, extension, supination, and pronation. These metrics are crucial for evaluating the restoration of elbow functionality and are measured using a goniometer, ensuring precision and reliability in the assessment of patient recovery.

### Complications

Recognizing that the path to recovery may be punctuated by various challenges, the form includes a section dedicated to documenting complications that may arise during the post-surgical rehabilitation process. This section encompasses:

- Instability, indicating a lack of joint security possibly affecting movement precision and control.
- Stiffness, reflecting reduced joint mobility which can severely impact daily activities and overall quality of life.
- Pain, a critical indicator of the patient’s comfort level and a potential obstacle to effective rehabilitation.
- Nervous damage, which can result from surgical interventions or the trauma itself, affecting sensory and motor functions of the arm.
- Other, a provision for recording any additional complications not previously categorized, ensuring a comprehensive capture of the patient’s post-operative journey.

### Patient selection in the study

Fifteen patients (Table 1) who underwent elbow surgery following trauma from May 16, 2019, to August 26, 2019, were selected and followed up at orthopedic clinics with weekly sessions dedicated to the upper limb. Patients not operated at this center and those operated at this location but did not perform follow-up checks at the same were excluded. The patient group included nine females and six males, all adults (skeletally mature), aged between 21 and 82 years, with an average age of 56 years. The selected patients were divided into groups by pathology type (fracture associated with dislocation and without) and subsequently whether a rehabilitative treatment was performed within the first 6-week post surgery. Ten patients with stable fracture (three radial head fractures, one radial and coronoid head fracture, four olecranon fractures, one radial and olecranon head fracture, and one distal humerus fracture) and five patients with unstable fracture (three radial head fractures, one distal humerus and radial head fracture, and one coronoid and radial head fracture) were selected. No distinction was made for the different types of fractures because it was not relevant from a rehabilitative standpoint as both bone structures and soft tissues are treated surgically. Of these 15 patients, five underwent surgical osteosynthesis and retensioning via anchor of the damaged ligament in patients with unstable fracture. The remaining 10 patients underwent osteosynthesis with plates and screws/cerclage/Kirschner wires. All patients selected in the study underwent X-rays before and after the operation and before the follow-up check-up on the AP and lateral planes.

**Table 1:** Patient descriptive statistics

Category	Value
Total number of patients	15
Patients with unstable fracture	5
Patients with stable fracture	10
Average age (years)	56
Age range	21–82
Number of male patients	6
Number of female patients	9

This table provides an overview of the study sample, including the total number of participants, the distribution of fractures (stable vs. unstable), the average age, the age range, and the gender distribution

### Post-operative

Selected patients were immobilized depending on the type of lesion and type of intervention. In this study, all patients were immobilized for a time equal to or less than 15 days.

### Clinical evaluation

Patients were then followed up in clinics for 45-day post operation, undergoing follow-up checks at 15, 30, and 45 days with a physical examination. X-rays conducted on the same morning were also reviewed during the second check-up. Joint ROMs were measured using a goniometer (see introduction and goniometric measurement). The flexion–extension ranges of motion were evaluated from the first follow-up, while pronation-supination was not always measurable due to the block in pronation-supination if indicated by the orthopedic. Goniometers and physical examinations were used as tools for measuring outcomes.

### Statistical analysis

Parametric tests, such as the Student’s T-test, were used to assess the significance of the average increase in joint ROMs recorded between the population who underwent rehabilitation and those who recovered only through an invitation to spontaneous mobilization. The study received approval from our institutional review board. All participants provided informed consent in accordance with our institution’s data collection and disclosure policy. Further ethical review was deemed unnecessary as no personally identifiable information was collected or stored.

## Results

The focus was on evaluating the impact of early physical therapy (PT) intervention—initiated within the first 6-week post surgery—on the recovery of joint range of motion (ROM) and the incidence of complications (Table 2). The follow-ups were scheduled at 15-, 30-, and 45-day post surgery to track the progression in ROM and note any emergent complications. This analysis divides the patients into two main categories based on the stability of the fracture: stable and unstable, with further subdivisions based on the receipt of physical therapy.

**Table 2:** Summary table of joint ROM changes across follow-ups

Category	Flexion (Avg ΔT1–T3)	Extension (Avg ΔT1–T3)	Pronation (Avg ΔT1–T3)	Supination (Avg ΔT1–T3)	Notes
Unstable fracture (Rehab Yes)	+ 20°	+ 60°	+ 40° (from T2)	+ 40° (from T2)	CRPS noted in 1 patient
Unstable fracture (Rehab No)	+ 10°	+ 20°	+ 45° (from T2)	+ 40° (from T2)	Stiffness complication in 1 patient
Stable fracture (Rehab Yes)	+ 30°	+ 30°	+ 40°	+ 30°	Complications in 3 patients (edema, stiffness, pain)
Stable fracture (Rehab No)	+ 20°	+ 10°	+ 60°	+ 40°	Stiffness complication in 1 patient

This table summarizes the average changes in joint ROM for patients analyzed in the study, differentiated by fracture type (stable vs. unstable) and participation in a rehabilitation program. The values expressed as “Avg ΔT1–T3” represent the average change in movement degrees from the initial follow-up (T1) to the end of the study (T3). For pronation and supination, changes are calculated from the second follow-up (T2) since not all patients were assessed for these movements at T1

### First follow-up (15-day post surgery)

At the first follow-up, data from 12 out of 15 patients were analyzed (three missed this appointment). None of the patients had started rehabilitation therapy by this point. The following summarizes the ROM findings and complications observed:

#### Patients with unstable fracture

- Participation: three out of five patients with unstable fractures were assessed.
- Complications: one patient developed complex regional pain syndrome (CRPS) in the hand.
- ROM Findings
- Flexion ranged from 110° to 120°.
- Extension deficits varied, with one patient having a − 80° measurement indicating severe limitation.

#### Patients with stable fracture

- Participation: ROM was measured in nine out of 10 patients with stable fractures.
- Complications: five patients reported pain and stiffness.
- ROM findings
- Flexion ranged from 90° to 110°.
- Extension deficits were observed, ranging from 0° to − 50°, indicating varying degrees of limitation.

### Second follow-up (30-day post surgery)

By the second follow-up, 14 patients were evaluated. They were divided into groups based on the initiation of physical therapy. Significant improvements were observed in those who underwent PT, though all patients showed progress in ROM:

#### Patients with unstable fracture

- Rehabilitation group: two patients showed improved flexion and extension, with one still experiencing CRPS.
- No Rehabilitation group: two patients did not undergo PT; one showed significant rigidity.

#### Patients with stable fracture

- Rehabilitation group: notable improvements in ROM, particularly in flexion and extension, were observed in patients who started PT between the 2nd- and 4th-week post surgery.
- No rehabilitation group: patients also showed improvement in ROM, albeit less pronounced than the rehabilitation group.

### Third follow-up (45-day post surgery)

This final follow-up showed continued improvement across all patients, with those undergoing PT generally displaying greater enhancements in ROM:

#### Patients with unstable fracture

- Continued improvement was noted, with patients undergoing PT showing the most significant recovery in both flexion and extension.

#### Patients with stable fracture

- Early rehabilitation: patients starting PT early showed the highest improvements in ROM, particularly in flexion and supination.
- Late rehabilitation: patients beginning PT between the 4th and 6th week also demonstrated significant recovery, albeit slightly less than the early rehabilitation group.
- No rehabilitation: continued stiffness was reported in some patients, indicating a slower recovery rate.

This study aimed to quantitatively evaluate the impact of rehabilitative intervention on the restoration of joint range of motion (ROM) in patients following surgical treatment for elbow fractures. The patient cohort was categorized based on the stability of the fracture (stable vs. unstable) and whether they participated in a rehabilitation program. ROM measurements—flexion, extension, pronation, and supination—were taken at three post-operative intervals: 15 days (T1), 30 days (T2), and 45 days (T3) after surgery.

### Methodological approach

The statistical methodology involved calculating the mean changes in ROM between each follow-up period (ΔT1–T2, ΔT2–T3, and ΔT1–T3) across the identified movements. The Shapiro–Wilk test verified the normality of ROM change distributions, supporting the application of the Student’s *t*-test for comparing the rehabilitation and non-rehabilitation groups. A significance level was set at *p* < 0.05.

### ROM changes across follow-ups

The analysis did not reveal statistically significant differences in mean ROM improvements between patients who underwent rehabilitation and those who did not, for all movements assessed during the intervals T2, T3, and T2–T3. Despite this, descriptive statistics highlighted trends toward improved outcomes in the rehabilitation cohort.

Notably, in patients with stable fractures, a comparative examination between the first and second follow-ups demonstrated a 20% increase in flexion ROM among rehabilitated patients, versus a 10% increase observed in their non-rehabilitated counterparts. Extension movements in the same group indicated a 40% improvement for rehabilitated patients, doubling the 20% increase seen in the non-rehabilitation group. Moreover, a distinct analysis of flexion ROM from T1 to T3 revealed varying degrees of improvement: patients initiating rehabilitation early (between T2 and T4 weeks) experienced a 37% increase, those beginning rehabilitation later (between T4 and T6 weeks) saw a 20% increase, and the non-rehabilitated group had a 15% increase.

### Clinical implications

While the statistical analysis did not identify significant differences in ROM recovery between the examined groups, the observed trends suggest a potential benefit of early rehabilitation in enhancing functional outcomes post-elbow fracture surgery. The absence of significant findings may be attributed to the limited sample size or variable individual responses to rehabilitation. The results underscore the importance of considering early rehabilitative interventions as part of post-operative care for elbow fractures. Future studies with larger patient cohorts and a more extended follow-up period are recommended to substantiate these findings and further elucidate the role of rehabilitation in post-surgical recovery.

## Discussion

In the exploration of rehabilitation’s efficacy following elbow fracture surgeries, our study ventured into the nuanced terrains of post-operative recovery, juxtaposing patients with stable and unstable fractures against the backdrop of their engagement in rehabilitation [18–21]. Through this lens, we aimed to uncover the extent to which rehabilitative interventions influence the restoration of joint range of motion (ROM) and mitigate post-surgical complications. The cohort characterized by its diversity in fracture stability and the binary of rehabilitative intervention—those who underwent rehabilitation versus those who did not—served as a fertile ground for analysis. In dissecting the outcomes among patients with unstable fractures, the revelation was stark; the anticipated disparities in recovery outcomes between the rehabilitated and non-rehabilitated were notably absent. This observation challenged the conventional wisdom, suggesting that rehabilitation, while beneficial under certain circumstances, is not a universal panacea for ensuring superior recovery trajectories [10, 13, 14, 22]. Conversely, the narrative among patients with stable fractures painted a slightly different picture. Despite the lack of significant differences in ROM improvements across all groups, a silver lining emerged in the form of complication resolution. Patients engaging in early rehabilitation showed notable progress in overcoming movement deficits, hinting at rehabilitation’s potential not just as a mechanism for expedited functional recovery but as a critical tool for holistic post-operative care, especially in circumventing the specter of stiffness and other movement impediments [14]. The limitations of our study—ranging from a constrained sample size to the heterogeneity in trauma types and rehabilitative treatments—cast a shadow over the generalizability of our findings. Coupled with a relatively short observation window, these constraints beckon for a cautious interpretation of the results, advocating for a broader and more nuanced understanding of the rehabilitation process in the post-surgical milieu. As we pivot toward future research, the call for a more homogenized study population and standardized rehabilitation protocols becomes louder [13, 14, 23, 24]. Such endeavors aim not only to sharpen the focus on rehabilitation’s role in surgical recovery but also to pave the way for evidence-based optimizations of post-operative care strategies. The aspiration for expanded patient cohorts and extended monitoring periods underlines the commitment to untangling the complexities of rehabilitation, ensuring that future investigations can offer more definitive conclusions. In conclusion, our study, navigating through the intricate dynamics of post-surgical recovery in elbow fracture patients, underscores the nuanced role of rehabilitation. While not indispensably linked to functional recovery in all instances, rehabilitative care emerges as a vital component in managing post-operative complications and enhancing patient outcomes. The insights gleaned beckon for a tailored approach to rehabilitation, one that is finely attuned to the individual patient’s needs, the specific nature of the fracture, and the overarching goal of restoring optimal functionality. As we advance, the fusion of empirical evidence with clinical prudence will undoubtedly illuminate the path to more effective, patient-centric post-operative care paradigms.

## Conclusions

Our investigation into the impact of rehabilitation on post-surgical recovery for elbow fracture patients underscores a nuanced landscape where rehabilitative care plays a crucial yet not universally definitive role in enhancing functional outcomes. Despite the absence of statistically significant differences in joint range of motion (ROM) improvements between patients undergoing rehabilitation and those who did not, early rehabilitative interventions showed potential benefits in mitigating post-operative complications, particularly stiffness. These findings highlight the importance of a tailored approach to post-operative care, emphasizing early intervention and patient-specific needs. The study’s limitations, including its small sample size and the heterogeneity of cases, suggest caution in generalizing the results and call for further research with larger cohorts and standardized protocols. Ultimately, our study contributes to the ongoing dialog on optimizing recovery strategies post-elbow fracture surgery, advocating for an evidence-based, individualized approach to rehabilitation.
